# 

**Published:** 1892-02

**Authors:** 


TO CONTRIBUTORS.

Articles for publication must reach this office not later than 15th inst.; and are expected to be published exclusively in this JOURNAL. Extra copies of JOURNAL furnished contributors when requested. Reprints by special arrangement. Publication of an article does not necessarily imply endorsement of the views therein expressed.

ATLANTA MEDICAL AND SURGICAL JOURNAL, Post-office box 431.

### SPECIAL NOTICE.

The office of THE JOURNAL will hereafter be with that of Dr. Hutchins, rooms 43 and 44 Old Capitol Building.

THE JOURNAL will be glad to have its friends, the doctors, call when in the city. Take the elevator, on Forsyth street side.

### A CHANGE IN PAPER.

Owing to our having changed the style of paper, upon which THE JOURNAL is printed, from a rough finish, *thick* paper to a *thinner*, glossy paper, which gives a better typographical display, the impression may exist that we have reduced the number of reading pages or that advertisements have decreased. SUCH IS NOT THE CASE. We still give you the full (64) *sixty-four* pages of reading matter and the advertising pages have *not decreased*. *Subscription*, $2.00 *per annum*.

